# The interplay between the airway epithelium and tissue macrophages during the SARS-CoV-2 infection

**DOI:** 10.3389/fimmu.2022.991991

**Published:** 2022-10-06

**Authors:** Emilia Barreto-Duran, Artur Szczepański, Adrianna Gałuszka-Bulaga, Marcin Surmiak, Maciej Siedlar, Marek Sanak, Zenon Rajfur, Aleksandra Milewska, Marzena Lenart, Krzysztof Pyrć

**Affiliations:** ^1^ Virogenetics Laboratory of Virology, Malopolska Centre of Biotechnology, Jagiellonian University, Krakow, Poland; ^2^ Department of Clinical Immunology, Institute of Pediatrics, Jagiellonian University Medical College, Krakow, Poland; ^3^ Department of Internal Medicine, Jagiellonian University Medical College, Krakow, Poland; ^4^ Astronomy and Applied Computer Sciences, Institute of Physics, Jagiellonian University, Krakow, Poland

**Keywords:** SARS-CoV-2, human airway epithelium (HAE), air-liquid interface (ALI), macrophages, co-culture, 3D cultures

## Abstract

The first line of antiviral immune response in the lungs is secured by the innate immunity. Several cell types take part in this process, but airway macrophages (AMs) are among the most relevant ones. The AMs can phagocyte infected cells and activate the immune response through antigen presentation and cytokine release. However, the precise role of macrophages in the course of SARS-CoV-2 infection is still largely unknown. In this study, we aimed to evaluate the role of AMs during the SARS-CoV-2 infection using a co-culture of fully differentiated primary human airway epithelium (HAE) and human monocyte-derived macrophages (hMDMs). Our results confirmed abortive SARS-CoV-2 infection in hMDMs, and their inability to transfer the virus to epithelial cells. However, we demonstrated a striking delay in viral replication in the HAEs when hMDMs were added apically after the epithelial infection, but not when added before the inoculation or on the basolateral side of the culture. Moreover, SARS-CoV-2 inhibition by hMDMs seems to be driven by cell-to-cell contact and not by cytokine production. Together, our results show, for the first time, that the recruitment of macrophages may play an important role during the SARS-CoV-2 infection, limiting the virus replication and its spread.

## Introduction

The SARS-CoV-2 caused a global pandemic of COVID-19 that killed millions and infected a large part of the global population (1). The disease starts as a viral infection that spreads through the respiratory tract, causing viral pneumonia of varying severity and systemic inflammatory response. The last phase of the disease, which is the most lethal (2), is characterised by an inappropriate, excessive and delayed triggered immune response that causes acute respiratory distress syndrome (ARDS) and the cytokine storm syndrome (CSS) (3, 4). Although the altered immune response in COVID-19 has been well described, the pathophysiology related to it and the alteration of immune cell functions during the infection is to be understood.

In the lungs, the first line of antiviral immunity is surveilled by cells that mediate innate immune responses, including mononuclear phagocytes (MNPs), such as monocytes, airway macrophages (AMs), and dendritic cells (DC) (3, 5–8). They are responsible for the phagocytosis of pathogens and recruitment of other cells through antigen presentation and cytokine release (3, 5, 6, 9). AMs play a dual role during viral infection, driving a pro-inflammatory response and combating the pathogen or supporting tissue repair and reducing the inflammation process (6, 9, 10). During viral infections, there is an increased infiltration of AMs in the lungs, in some cases, resulting in an exaggerated pro-inflammatory response and tissue damage (11–15).

AMs can be categorized as resident or monocyte-derived macrophages (hMDMs) depending on their origin (3, 6). Resident macrophages originate from embryonic precursors and localize near the epithelial layer of the alveoli and conductive airways (6). The hMDMs differentiate from the monocytes, migrating from the blood to the airway lumen by transepithelial motility (6, 16–18). Depending on their role and activation status, macrophages can be classified as M1 or M2 (3, 8, 9, 19, 20). M1 macrophages are capable of pro-inflammatory responses and production of pro-inflammatory cytokines such as IL-6, IL-12 and tumour necrosis factor-alpha (TNF-α). In contrast, M2 macrophages have anti-inflammatory activity and promote the repair of damaged tissue (21–24). AMs express the SARS-CoV-2 receptor, angiotensin-converting enzyme 2 (ACE2), and are susceptible to infection (25–29). Previous reports proposed that AMs can act as “Trojan horses” and help spread the infection by carrying the virus to other tissues (30, 31). On the other hand, only abortive infection was recorded for these cells, indicating viral entry but a lack of efficient viral replication or infectious particles release (25, 26, 32–34). Further, no direct evidence of the cell-cell transmission of SARS-CoV-2 from hMDMs to other permissive cells was shown (31, 33, 35). What is more, most of the studies of AMs’ role in the course of COVID-19 focused on either clinical lung samples or *in vitro* analysis of isolated cell populations, while little is known about the virus-host cell interplay at the site of infection.

Hence, we studied the role of macrophages during the SARS-CoV-2 infection, using an *ex vivo* 3D fully differentiated human airway co-culture model. We showed, for the first time, that the presence of hMDMs modulates the SARS-CoV-2 replication in the co-culture comprised of human airway epithelium (HAE) and hMDMs. Together with the confirmation of hMDMs abortive infection and the lack of viral transmission by infected macrophages, this indicates a protective role of AMs at the site of the infection.

## Materials and methods

### Monocyte isolation and differentiation

Anticoagulated citrate dextrose-A-treated blood from healthy donors was purchased from the Regional Center of Blood Donation and Blood Therapy in Krakow, Poland. Peripheral blood mononuclear cells (PBMC) were isolated by the standard Ficoll/Isopaque (Pharmacia, Sweden) density gradient centrifugation. Monocytes were then separated from PBMC with the AVANTI J-26S XP elutriation system, equipped with the Sanderson separation chamber (Beckman, USA), as described previously (36). Isolation purity was over 95% as tested by staining with anti-CD14 mAb (BD Biosciences Pharmingen, USA) and flow cytometry analysis (FACSCanto flow cytometer, Becton Dickinson, USA). Cells were washed and resuspended in RPMI 1640 medium (Thermo Fisher Scientific, USA) and kept in an ice bath until used.

Monocytes obtained by elutriation were differentiated for 8-10 days to hMDMs (37). Three million monocytes per well were seeded in 6-well plates (Ultra-Low Attachment (ULA) Multiple Well Plate, Corning^®^ Costar^®^, USA) with RPMI medium supplemented with 10% LPS free Foetal Bovine Serum (FBS, Biowest, France). The medium was changed every 48 hours. On days 8-10, cells were detached using ACCUTASE™ (Stemcell Technologies, USA) and the phenotype profile was performed by immunostaining. The macrophages differentiation markers CD68, CD80 and CD163 were analysed (**Supplementary Figure 1**).

### Human airway epithelial cultures

Primary human bronchial epithelial cells were purchased (Epithelix Sarl, Switzerland) and expanded in bronchial epithelial growth medium (BEGM) in-house. When confluent, cells were detached using trypsin and seeded onto permeable Thincert™ culture inserts (Greiner Bio-One, cat. no. 662641, Austria). Cells were cultured submerged in BEGM medium on the apical and basolateral side until confluent, next the apical medium was discarded, while the basolateral medium was changed to an air-liquid interface (ALI) medium. Cells were cultured for 4 weeks to form fully-differentiated, polarized cultures that manifested *in vivo* pseudostratified mucociliary epithelium phenotype. Commercially available MucilAir™-Bronchial (Epithelix Sarl, Switzerland) HAE cultures were also used in some experiments. MucilAir™ cultures were maintained as suggested by the provider, in MucilAir™ culture medium. All cells were maintained at 37°C under 5% CO_2_.

### HAE-hMDMs co-cultures

The commercially available MucilAir (Epithelix, France) or the in-house produced HAEs were used for the preparation of co-cultures with primary hMDMs. The hMDMs (infected or not infected) were detached from the ultra-low attachment plates and seeded on the apical or basolateral side of the HAEs, before or after the infection as described above.

To obtain the apical co-culture, 100,000 macrophages were seeded onto the apical side of HAE. The HAEs with hMDMs were incubated for 4 hours at 37°C, next, the apical medium was removed and cultures were maintained at ALI conditions. To obtain the basolateral co-culture, inserts were inverted and placed in a Petri dish with 2 ml of PBS (to prevent drying); then, the hMDMs were inoculated on the basolateral side of the membrane. The dish was closed and cultured for 4 hours at 37°C. After the incubation, inserts were reverted to their original position, leaving the hMDMs submerged in the basolateral medium.

To obtain co-cultures with apical and basolateral hMDMs, half of the hMDMs were added on the basolateral side, as described above, and incubated for 2 hours. Next, the inserts were reverted to the original position and the other half of the hMDMs were inoculated onto the apical side and incubated for another 2 hours, as described above.

### Viral infection of hMDMs

hMDMs were infected for 2 hours at 37°C with SARS-CoV-2 (clinical isolate PL1455, GISAID accession number: EPI_ISL_451979) at 5000 TCID_50_/ml. After the infection, hMDMs were either washed thrice with PBS or acid-washed with low pH buffer (1% acetic acid + 25% NaCl (4 M) in ddH_2_O). Acid-wash samples were first washed once with PBS, then incubated for 1 minute with the low pH buffer and washed the last time with PBS. Acid-washed cultures were used as controls, to ensure that no infectious particles were present on the cell surface, either bound to the cell membrane or suspended in the supernatant. For co-culture experiments, hMDMs were detached after washing and resuspended in RPMI supplemented with 5% FBS for further co-culture with HAE.

### Infection of HAE in monocultures or co-cultures

HAE and HAE-hMDMs co-cultures were apically infected for 2 hours at 37°C with SARS-CoV-2 with a TCID_50_ of 5000/ml or mock infected. After infection, cultures were washed thrice with PBS, and the third wash PBS portion was collected as a 2 h p.i. sample. If hMDMs were to be added after the infection, the HAEs were inoculated with the virus or mock, washed, the 2 h p.i. samples collected, and then the hMDMs were added. After the addition of the hMDMs the cultures were maintained at 37°C for 4 hours before living them at ALI. All cultures were left in ALI for the remaining time of the experiment. Later, samples were collected every 24 hours until 120 h p.i. For sample collection, PBS (100µl) was added daily to the apical side of the inserts and incubated for 15 minutes at 37°C, then it was collected for RT-qPCR analysis.

### hMDMs viral transfer experiments

The hMDMs were infected for 2 hours at 37°C with SARS-CoV-2 with a TCID_50_ of 5000/ml or mock-infected. After infection hMDMs were washed trice with PBS, or acid-washed. The third wash PBS portion was collected as 2 h p.i. sample. Infected hMDMs were detached and seeded on the apical side of the HAE, and co-cultures were incubated overnight. At 24 h p.i. the remaining apical medium was removed. Next, PBS was added to the apical side of the inserts, incubated for 15 minutes at 37°C and collected for RT-qPCR analysis. Samples for RT-qPCR analysis were collected daily, as described previously.

### Isolation of nucleic acids, reverse transcription and quantitative PCR

Viral RNA was isolated from cell culture supernatants and cell lysates using a Viral DNA/RNA Kit (A&A Biotechnology, Poland), according to the manufacturer’s instructions. Isolated viral RNA was quantified using GoTaq^®^ Probe 1-Step RT-qPCR System (Promega, USA). Specific SARS-CoV-2 probe (200 nM, ACT TCC TCA AGG AAC AAC ATT GCC A (FAM/BHQ1) and primers (Forward: 600 nM, CAC ATT GGC ACC CGC AAT C, Reverse: 800 nM, GAG GAA CGA GAA GAG GCT TG) were used for the RT-qPCR reaction. The heating scheme was: 15 min at 45°C and 2 min at 95°C, followed by 40 cycles of 15 s at 95°C and 1 min at 58°C or 60°C. In order to determine the copy number of the virus N gene, corresponding DNA standards were prepared and serially diluted.

### Cytokine analysis

Each sample collected every 24 hours from the basolateral medium of the co-cultures was stored at -80°C for further analysis. Assessment of levels of selected proteins was performed with the use of xMAP technology Luminex assay (Human XL Cytokine Luminex Performance Assay 44-plex Fixed Panel (LKTM014), Bio-Techne, Minneapolis, MN, USA) and MAGPIX fluorescent-based detection system (Luminex, Austin, TX, USA) according to the manufacturer’s protocol. All results were interpreted from the calibration curves in pg/ml.

### Immunostaining

Cells and co-cultures were fixed for 1 hour with 4% PFA and washed with 1× PBS. Before staining, samples were permeabilized with 0.5% Triton X-100 for 20 min (HAE-hMDMs co-cultures) or 1 min (hMDMs) at room temperature (RT), washed with 1×PBS and blocked with 10% BSA for 1 hour at 37°C (HAE-hMDMs co-cultures) or with 5% BSA for 30 min at RT (hMDMs). After blocking, primary antibodies (**Table 1**) diluted in BSA 1% were added and incubated overnight at 4°C. Then, primary antibodies were washed thrice with PBS supplemented with 0.5% Tween. Secondary antibodies, diluted in 1% BSA, were added and incubated for 2 hours (for HAE-hMDMs co-cultures) or 1 hour (hMDMs) at RT. The excess of secondary antibodies was washed thrice with PBS supplemented with 0.5% Tween followed by 30 minutes of incubation with phalloidin and DAPI for actin filaments and nuclei staining, respectively. Finally, cells were washed trice with 1×PBS and mounted in glass slides with ProLong™ Diamond Antifade Mountant (Thermo Fisher Scientific, USA). For co-cultures, the insert membrane was cut with a scalpel prior to mounting. Fluorescent images were acquired using Carl Zeiss, ZEN 2012 SP1, LSM 710 confocal microscope (Carl Zeiss Microscopy GmbH).

**Table 1:** Antibodies used for immunostaining.

Antibody	Host specie	Dilution and final concentration	Company (Catalog #)
Anti-SARS CoV-2 Nucleocapsid	Rabbit	1:200 (5µg/ml)	Invitrogen (MA5-36251)
Anti-SARS CoV-2 Nucleocapsid	Mouse	1:200 (5µg/ml)	Invitrogen (MA5-29981)
CD68/SR-D1	Mouse	1:50 (10µg/ml)	R&D Systems (MAB20401)
CD68	Mouse	1:100 (5µg/ml)	Invitrogen (14-0688-82)
CD80 (B7-1)	Rabbit	1:100 (10µg/ml)	Invitrogen (PA5-85913)
CD163	Mouse	1:100 (10µg/ml)	OriGene (TA506391)
Anti-rabbit Alexa Fluor 488 Secondary antibody	Donkey	1:400 (5µg/ml)	Invitrogen (A-21206)
Anti-rabbit Alexa Fluor 488 Secondary antibody	Goat	1:400 (5µg/ml)	Invitrogen (A-11034)
Anti-mouse Alexa Fluor 488 Secondary antibody	Donkey	1:400 (5µg/ml)	Invitrogen (A-21202)
Anti-rabbit Alexa Fluor 594 Secondary antibody	Goat	1:400 (5µg/ml)	Invitrogen (A-11012)
Anti-mouse Alexa Fluor 546 Secondary antibody	Donkey	1:400 (5µg/ml)	Invitrogen (A-10036)
Anti-rabbit Alexa Fluor 546 Secondary antibody	Goat	1:400 (5µg/ml)	Invitrogen (A-11035)
Rabbit IgG isotype control	–	Same concentration as used for primary antibody	GeneTex (GTX35035)
Mouse IgG isotype control	–	Same concentration as used for primary antibody	GeneTex (GTX35009)

### Statistical analysis and image processing

Statistical analysis was performed using GraphPad Prism 7 software (GraphPad Software Inc., San Diego, CA). Statistical significance was estimated using paired T-tests for two group comparison and Kruskal-Wallis test with Dunn’s *post-hoc* test for multiple groups comparison. The P values <0.05 were considered significant. Images obtained from the confocal microscope were processed in ImageJ Fiji (38).

## Results

### hMDMs undergo an abortive SARS-CoV-2 infection

hMDMs differentiation from primary human peripheral blood monocytes was confirmed based on their morphology and phenotype (**Supplementary Figure S1**). All hMDMs expressed monocytic lineage marker CD68, with a diverse expression of CD80 and CD163, the markers of M1 and M2 macrophages, respectively (19, 39) (**Supplementary Figure S2**).

**Figure 1:**
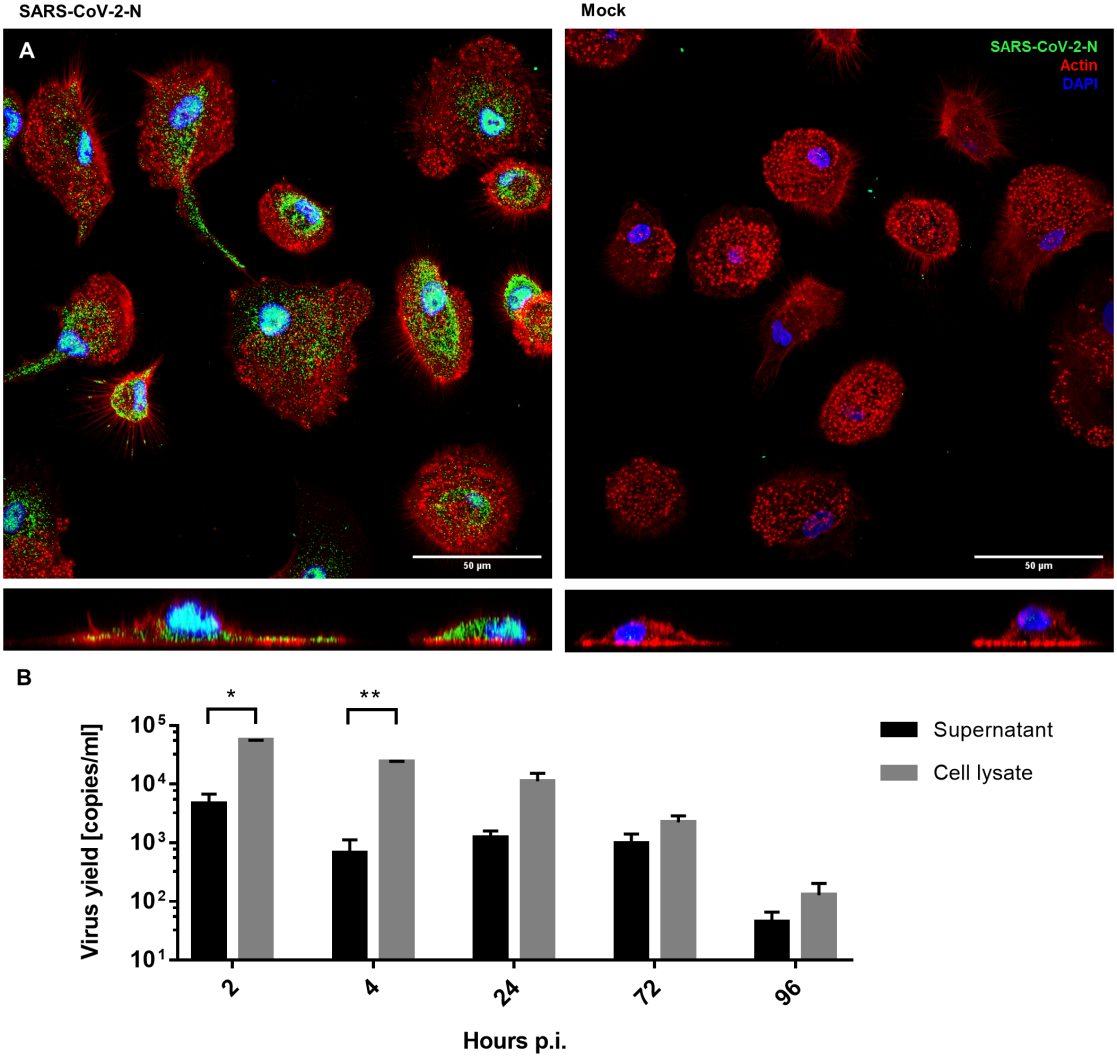
hMDMs infected with SARS-CoV-2 virus or inoculated with mock. **(A)** Confocal microscopy images of SARS-CoV-2-infected and mock-infected macrophages 48 h p.i., showing the virus N protein (green), actin (red), and nuclei (blue). The bottom panels show an orthogonal view of cells. **(B)** Virus yield (viral RNA copies/ml, as determined with RT-qPCR) in cell culture supernatants and cell lysates of infected hMDMs. The experiment was carried out in triplicate and means ± SEM are shown. Groups were compared by paired T-test. *p < 0.05. **p<0.01.

hMDMs were infected with SARS-CoV-2 (5000 TCID_50_/ml) or mock exposed for 2 hours and then cultured for 96 hours. The viral infection was evaluated using immunostaining for the viral nucleocapsid protein (NP) and RT-qPCR of viral genome. Immunostaining revealed that the cells were infected and expressed NP inside the hMDMs cytoplasm, while no specific NP signal was present in mock-infected cells (48 h p.i.) (**Figure 1A**). At the same time, no effective replication of viral RNA was observed, and the RT-qPCR analysis showed a gradual decrease in the number of viral RNA copies over time, both in the supernatant and in the cell lysate. This confirmed that there is no effective replication taking place (**Figure 1B**).

### hMDMs do not serve as virus carriers

**Figure 2:**
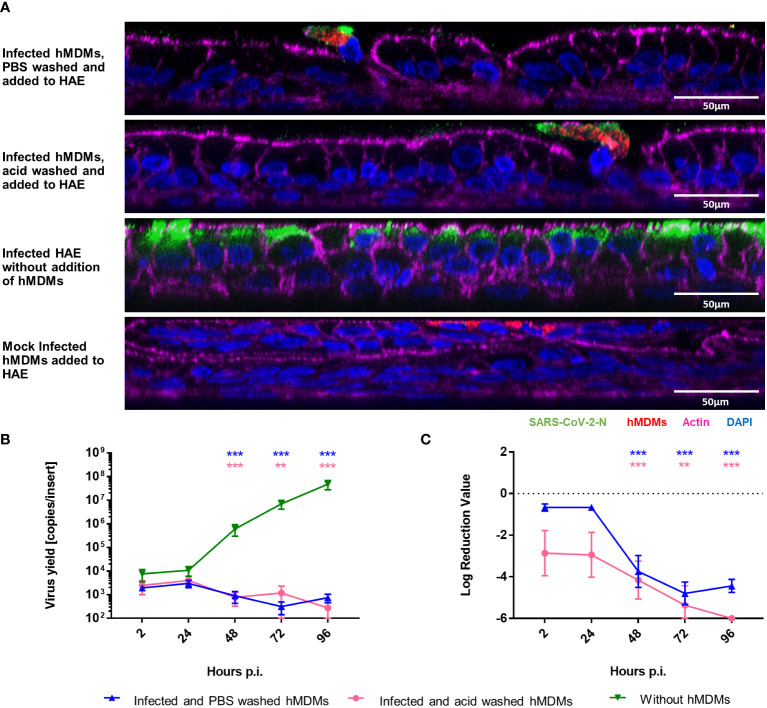
hMDMs do not transfer the infectious virus to other cells or tissues. SARS-CoV-2 Infected hMDMs were added to HAE to analyse the virus transmission from hMDMs to other cells. **(A)** Confocal microscopy images of HAE co-cultured with infected hMDMs were taken. Mock control and infected HAE without hMDMs are also shown. Images were obtained at 72 h p.i. SARS-CoV-2 N protein is shown in green; CD68, hMDMs marker, is shown in red, actin is shown in magenta and nuclei in blue. **(B, C)** graphs shows the quantification of viral replication in hMDMs co-cultures, evaluated by RT-qPCR. Data are presented as the number of RNA copies per ml **(B)** and the relative log reduction in the virus yield **(C)**. Data were obtained from three independent experiments, each experiment was carried out in triplicate and means ± SEM is shown. Groups were compared by the Kruskal-Wallis test and Dunn’s multiple comparisons test. **p<0.01, ***p<0.001.

Since we demonstrated that viral replication in hMDMs was not productive, we also wanted to determine if SARS-CoV-2 infected hMDMs can transport the virus to other permissive cells and, possibly, other tissues. For this purpose, we analysed the transfer of virions from infected hMDMs to the airway epithelial cells during the co-culture. First, hMDMs were infected and the unbound virus was removed by PBS washing. Next, the cells were treated with the low-pH buffer to inactivate the virions that were not internalized or washed again with PBS. Such pre-treated samples were then seeded onto the apical side of HAE cultures. The supernatants were collected every 24 h p.i. until 96 h p.i. next cells were fixed at different time points. Virus yields were quantified with RT-qPCR and the presence of virus proteins was evaluated by immunostaining. Mock-infected hMDMs were used as negative controls. Confocal images showed localization of the viral protein within hMDMs in the co-cultures after 72 h p.i., but no viral protein was observed in the epithelial layer, in contrast to infected HAE without hMDMs (**Figure 2A**). RT-qPCR results corroborated with the lack of viral replication in HAE, both in the only PBS-washed and acid-washed conditions (**Figures 2B, C**).

### hMDMs hamper virus replication in the ex-vivo HAE co-culture model

**Figure 3:**
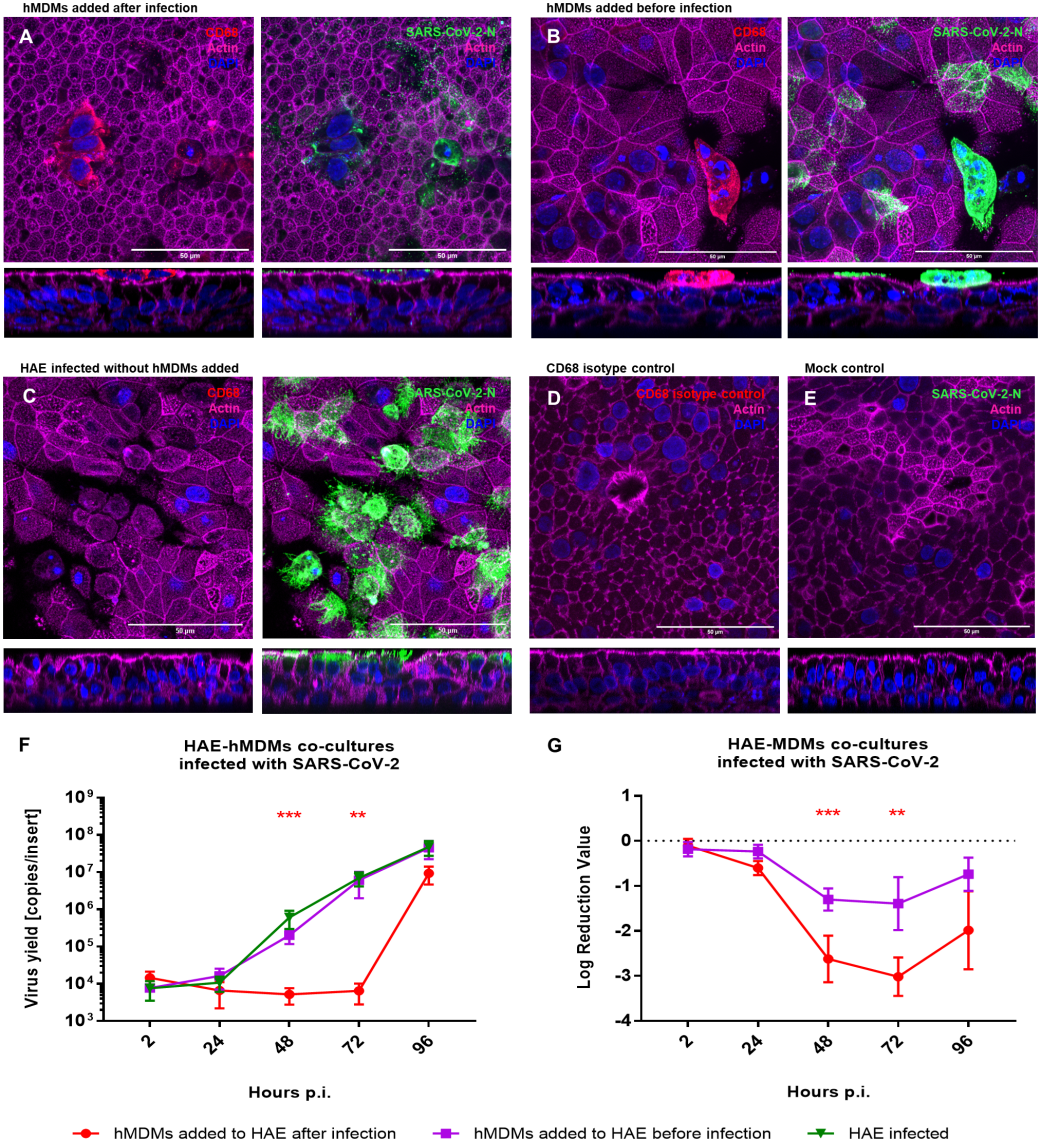
HAE-hMDMs co-cultures infected with SARS-CoV-2. hMDMs were added to the HAE before and after SARS-CoV-2 infection. **(A, B)** Confocal images of HAE-hMDMs co-cultures and **(C-E)** controls. Co-cultures were fixed 72 h p. i. and stained for CD68 (red left panels), virus (green right panels), actin (magenta) and nuclei (blue). Corresponding orthogonal views are shown below each image. **(F, G)** graphs showing the quantification of viral replication in co-cultures, evaluated by RT-qPCR. Data were obtained from three independent experiments, each experiment was carried out in triplicate and means ± SEM is shown. **(F)** shows virus yields expressed as RNA copies per millilitre, **(G)** shows the log reduction value of viral RNA in HAE-hMDMs co-cultures calculated using the values of the infected HAEs without hMDMs as value zero. Groups were compared by the Kruskal-Wallis test and Dunn’s multiple comparisons test. **p<0.01, ***p<0.001.

As we already observed that macrophages do not serve as virus carriers, we aimed to elucidate the role of hMDMs in the course of SARS-CoV-2 infection. For this purpose, we added hMDMs to the HAE culture, before or after the infection (2 h p.i.), and compared the viral replication in the co-cultures and monocultures. Mock-infected HAE and mock-infected co-cultures were used as negative controls. Viral replication was quantified by RT-qPCR, while localization of the viral NP within the cells or the virus spread was assessed by confocal microscopy (**Figure 3**). Viral RNA abundance or viral replication kinetics showed no difference between the HAE cultures infected alone or in the co-culture with hMDMs, if the latter were seeded before the infection (**Figures 3F, G**). However, seeding of the hMDMs onto infected HAEs had a strikingly different effect, because the infection was vastly inhibited during the first 72 h p.i. The difference reached as much as 3 log10 after 72 h p.i., and was statistically significant (**Figure 3G**). The confocal images of the co-cultures at this timepoint (72 h p.i.) showed that hMDMs were infected with the virus regardless of the time of addition of the hMDMs to the HAE (before or after HAE inoculation with the virus), however, hMDMs added before infection presented higher infection burden. Also, limited viral spread in the epithelial cells was observed when hMDMs were added after infection as compared with co-cultures where hMDMs were added before the infection, or with HAE infected without the addition of hMDMs (**Figures 3A, D**). Additionally, we evaluated phenotypes of hMDMs polarization in the co-cultures by the immunostaining of the differentiation markers CD80 and CD163. Immunostaining of co-cultures (samples fixed at 48 h p.i) revealed that the percentage of hMDMs expressing both markers with a predominant expression of CD163 (M2) is higher than in infected hMDMs in monoculture or in co-cultures, where the hMDMs were added to the basolateral side of the membrane (**Supplementary Figure S3**). Additionally, among the co-cultures, a higher expression of CD163 was shown in the co-culture condition where the hMDMs were added after the infection (**Supplementary Figure S3**).

### Apical localization of hMDMs in the HAE-hMDMs co-cultures is crucial to hamper the viral replication

**Figure 4:**
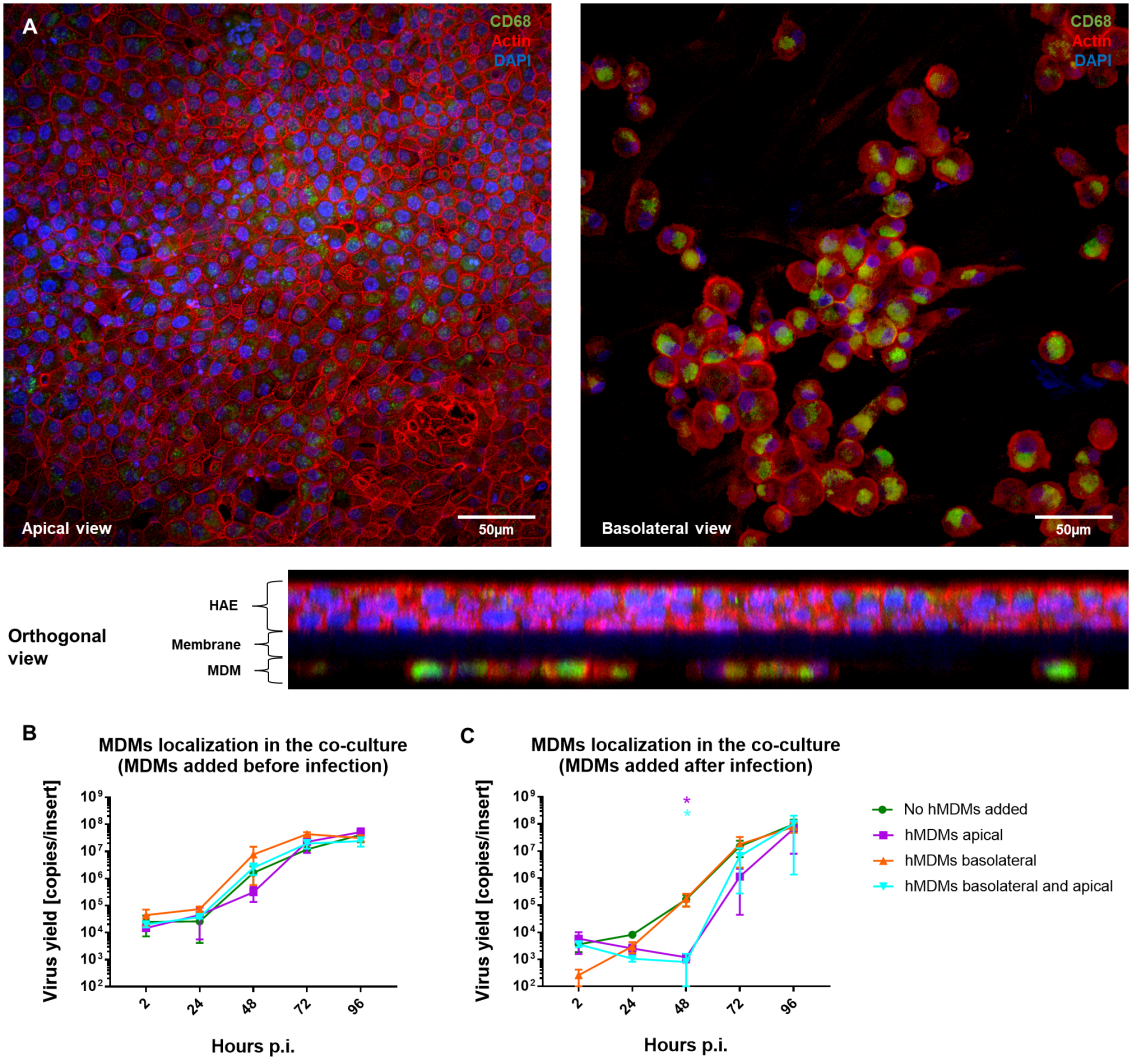
Comparison of the basolateral and apical placement of hMDMs in hMDMs-HAE co-cultures. hMDMs-HAE co-cultures where hMDMs were placed both apically and in the basolateral side. **(A)** Confocal images showing the apical, basolateral and orthogonal view of the co-cultures. HAE-hMDMs co-cultures were fixed 72 hpi and stained for CD68, hMDMs marker (green), actin (red) and DAPI (blue). **(B, C)** Quantification of viral replication in co-cultures, evaluated by qPCR. **(B)** Shows virus yields expressed as RNA copies per milliliter in co-cultures where hMDMs were added before infection and **(C)** after infection. Data were obtained from three independent experiments, each experiment was carried out in triplicate and means ± SEM is shown. Groups were compared by the Kruskal-Wallis test and Dunn’s multiple comparisons test. *p < 0.05.

Once we showed that the hMDMs are able to modulate the virus replication in the epithelial cells, the role of hMDMs localization in the co-culture was evaluated. For this purpose, hMDMs were seeded either on the apical or basolateral side of the HAE monolayer, or on both sides, and the effect on viral replication was analysed by RT-qPCR. The presence and localization of hMDMs in the co-cultures were confirmed by confocal microscopy. Following a co-culture for 96 h p.i., we were able to confirm the presence of hMDMs both on the apical side of the HAE cultures and on the basolateral side, attached to the insert membrane (**Figure 4A**). RT-qPCR results showed no viral replication inhibition when hMDMs were added only on the basolateral side of the membrane, and similarly to the previous experiments, no significant differences in the viral replication kinetics were observed when the hMDMs were added before the inoculation of the virus (**Figure 4B**). However, when hMDMs were added after infection and seeded apically, or on both sides of the epithelial layer, a significant delay in SARS-CoV-2 replication was observed (>2 log_10_ inhibition at 48 h p.i.) (**Figure 4C**).

### hMDMs viral inhibition does not dependent on the cytokine release in co-cultured cells

**Figure 5:**
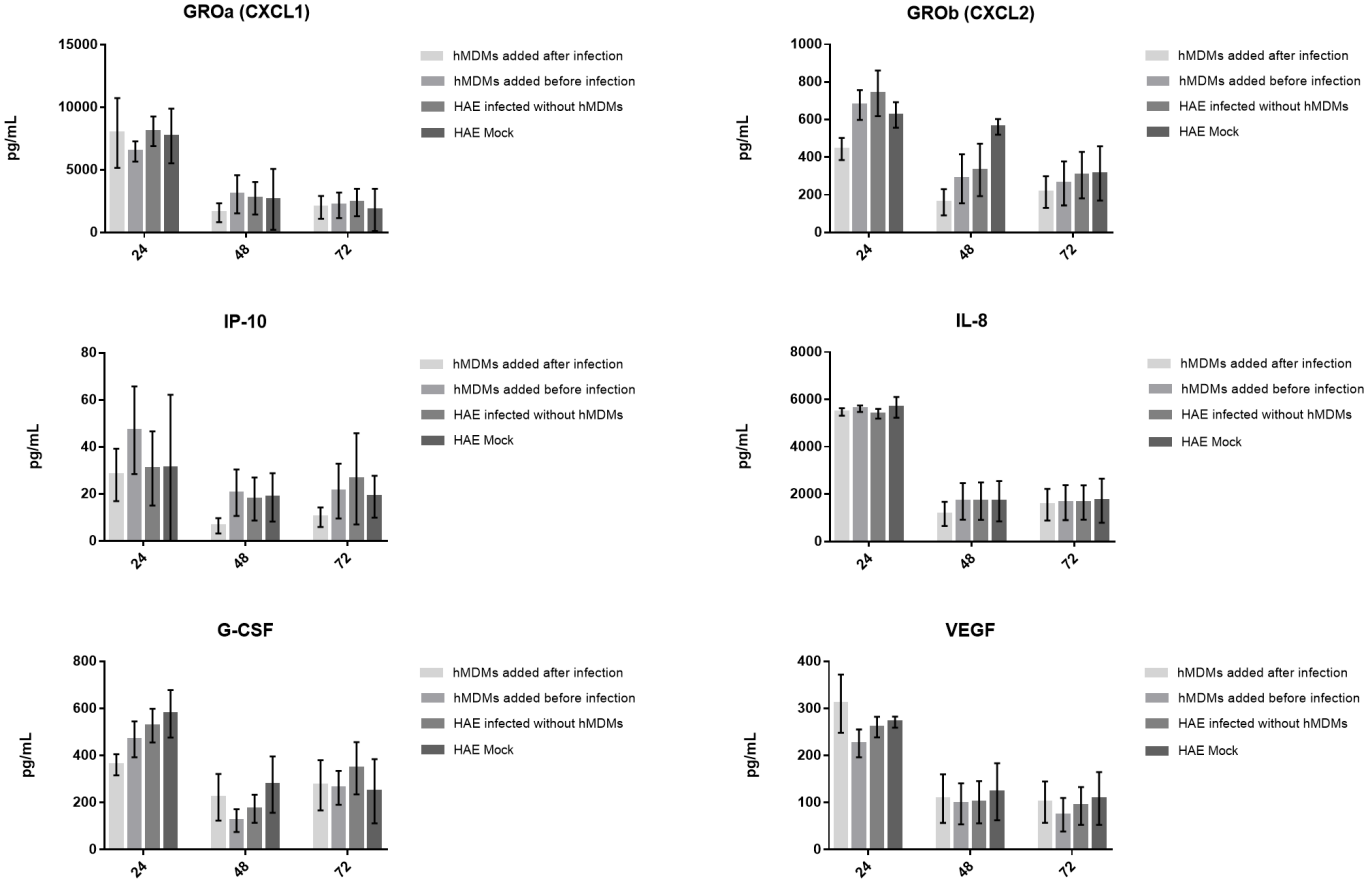
Production of cytokines and chemokines by the SARS-CoV-2 infected co-cultures. The graphs show the cytokine content in pg/ml present in the culture medium. Data was obtained from three independent experiments; each experiment was performed in triplicate and means ± SEM are shown. Groups were compared using Kruskal-Wallis test and Dunn’s multiple comparisons test.

Finally, to determine if cytokine production might be important for the hMDMs-mediated viral inhibition, we evaluated the levels of 44 cytokines and chemokines in the basolateral medium of the co-cultures. Samples were collected and analysed at 24, 48 and 72 h p. i. In all three experiments, the secretion of four chemokines (CXCL1, IL-8, CXCL2, IP-10) and two growth factors (G-CSF and VEGF) was detected (**Figure 5**). We noticed that CXCL2 and IP-10 levels were decreased after 24 h p. i. and 48 h p. i., respectively, but only if hMDMs were added after the inoculation of the virus. However, no statistically significant differences were observed.

## Discussion

The HAE model is one of the most relevant *ex vivo* models to monitor the events occurring at the microenvironment of the respiratory tract (40–42). HAEs are composed of different types of epithelial cells (basal, goblet, club, and ciliated), and show a phenotypic similarity with the *in vivo* respiratory epithelium, including the mucus production and barrier functions (43, 44). However, while HAEs are an advanced product of tissue culture, several components are missing, including the immune cells. The immune cells play an important role *in vivo*, in the resistance against infection, tissue modelling and signalling, and their deficiency can drastically alter tissue homeostasis (10, 17). Furthermore, it is known that the crosstalk between the epithelium and the immune cells plays a crucial role during viral infections (3, 5, 6, 9). This is why we aimed to develop a human airway co-culture model that incorporates not only the fully differentiated epithelial layer but also cells from the immune system, that permit us to verify the role of AMs in the course of SARS-CoV-2 infection *in vitro*, in conditions reassembling the natural ones the most.

In the first place, we infected hMDMs with SARS-CoV-2 to determine cell susceptibility to infection and viral replication. The results showed no productive infection in hMDMs, as the viral RNA copies were decreasing in time, both in cell supernatants and cell lysates. These results confirmed previously reported abortive SARS-CoV-2 infection of hMDMs (25, 26, 32–34, 45). Evidently, the virus could enter the hMDMs but was unable to produce a viral progeny. Similar observations were previously reported for SARS-CoV-1 (46–48). Thereafter, although SARS-CoV-2 abortive infection was confirmed, we wanted to determine if hMDMs were able to transfer the virus by cell-cell contact to other permissive cells. It has been demonstrated earlier that other viruses can infect monocytes/macrophages and use them as reservoirs and dissemination carriers to spread the infection to other tissues. Viruses such as the HCMV (49, 50), HIV-1 (51), HCoV-229E (52), among others (9, 53), infect AMs and have been proposed to use AMs as “Trojan horses” for propagation. A similar phenomenon was also suggested for SARS-CoV-2 (30, 31) and viral transmission from hMDMs to Vero E6 cell line has been shown by Lv et al. (35) and Percivalle et al. (31). However, in our study, by co-culturing infected hMDMs with non-infected HAE cultures we have shown that hMDMs were not able to transfer the virus to the airway epithelial cells and therefore were probably not responsible for viral dissemination *in vivo*. There may be several explanations for these contradictory results. First, different cell models were used in these studies. Both referred studies used AMs together with the Vero E6 cell line which is an animal-origin kidney cell line and may vary in biological characteristics (e.g. membrane proteins, cathepsin expression), compared to the primary airway epithelial cells. HAE cultures have physiological characteristics like mucus production, ciliation, and extended glycocalyx that can and will severely influence the cell-cell interaction. Next, Lv et al. used murine alveolar macrophages instead of human-origin AMs and showed the viral transmission from AMs to Vero E6 by using the supernatant of infected AMs. This is surprising, as the incapability of AMs to produce infectious progeny has been well demonstrated (25, 31–33), and may suggest model or protocol-related differences. On the other hand, Percivalle et al. demonstrated that AMs were able to transfer the virus to the Vero E6 by cell-cell membrane fusion. As aforementioned, the cell-cell transfer process is likely to differ for the fully differentiated tissues. However, it is also worth noting that the authors washed the hMDMs with Trypsin/EDTA before inoculation onto the VERO E6 cells (31). Considering the requirement of the Spike protein to be proteolytically activated, one may not reject the hypothesis that such treatment supported the cell-cell fusion (54). In our study, we washed hMDMs with an acidic buffer to wash off but also to disable the viral particles attached to the membrane. No viral transmission from the hMDMs to the HAE was observed.

Next, we wanted to verify the role of macrophages in the course of SARS-CoV-2 infection using the hMDMs-HAE co-culture model. We studied the influence of 1) the time of hMDMs addition (before or after the infection) and 2) localization of hMDMs in the co-culture (apical or basolateral side of the epithelial layer). The time of addition analysis showed substantial differences in viral replication. A delay in viral replication was observed when hMDMs were added after infection, compared to the condition when hMDMs were added before inoculation. These results indicate an antiviral activity of hMDMs, which may be lost during the viral infection, and thus, get hampered if hMDMs were infected together with the epithelial cells. This, suggests that during a natural infection with SARS-CoV-2 the protective role of the resident AMs might be inhibited. Further investigation regarding the loss of antiviral activity of hMDMs after SARS-CoV-2 infection is necessary to confirm our hypothesis. What is more, in the co-culture experiments evaluating the location of hMDMs on the epithelial layer, we found that not only the addition of hMDMs after the infection is crucial for the hMDMs-mediated viral inhibition but also a close contact of the hMDMs with the epithelial layer is necessary. This is also in agreement with our results for the M1/M2 polarization evaluation. The immunostaining results showed a predominant M2 polarization in the co-cultures where hMDMs were added apically compared with the infected monoculture of hMDMs or the co-culture condition where the hMDMs were added on the basolateral side, without direct contact with the HAE. This suggests a polarization of the hMDMs to M2 following the contact with epithelial cells. Additionally, the highest expression of CD163 (M2) was shown in the co-culture condition where the hMDMs were added after the infection (condition that showed a delay in the viral replication). Together these results suggest a relation between the antiviral effect of hMDMs and their polarization to M2 mediated by their contact with the epithelial cells.

Our model in which hMDMs were added before the infection can be associated with the response of resident AMs in the lungs, which are present *in situ* when the virus reaches the tissue. On the other hand, the hMDMs that are added after the infection reflect well an innate immune response *in vivo* consisting of migration of blood-derived monocytes differentiating next into macrophages at the site of infection (3, 6, 16–18). The confocal microscopy images of the co-cultures showed that both, hMDMs that were added before and after the infection, were infected since viral protein was detected in both cases. Nevertheless, taking into consideration the differences in viral replication between the co-cultures with hMDMs added before and after the infection, we suggest that the viral NP observed inside hMDMs may have originated from different sources. In the experiment where hMDMs were added before the infection, intracellular NP could be a consequence of direct infection of the hMDMs (resident AMs), while the viral protein inside hMDMs (migrating hMDMs) that were added after the infection might be of indirect origin as a result of the phagocytosis of the virus, of infected epithelial cells or cell debris. This is in agreement with previous reports indicating the presence of viral protein inside hMDMs as a result of either an ACE2-mediated entry (25, 27–29), viral particle phagocytosis (35) or phagocyted infected cells (55). The phagocytosis-mediated inhibition hypothesis is also in agreement with our finding on M2-mediated antiviral response. Thus, taking into account that the M2 phenotype is highly related to the phagocytic activity (56–58), we suggest that M2 polarized hMDMs that have contact with infected epithelial cells are responsible for controlling the viral spread by phagocyting and digesting the infected cells and the virus. On the other hand, the antiviral activity of hMDMs that were infected directly (in our model - added before infection) might be downregulated by their infection, resulting in an inhibition of hMDMs ability to control SARS-CoV-2 replication in the epithelial cells. Nevertheless, this should be confirmed with further experiments. Speranza et al. (55) proposed, based on a transcriptional analysis of cells recovered from SARS-CoV-2-infected nonhuman primates, that the high percentage of viral RNA positive macrophages they found, was the result of phagocytosis of infected cells. Interestingly, the authors also showed that infiltrating MDMs, were responsible for phagocytosis of infected cells early in the infection, rather than the resident macrophage population. In another study, Fujimoto et al. (59) showed that influenza virus-infected HeLa cells were phagocyted by macrophages that were added after the infection, resulting in complete inhibition of virus dissemination. Also, by placing the macrophages and the HeLa cells on opposite sides of a permeable membrane, the Authors proved that the secretion of antiviral soluble factors from the macrophages was not responsible for the viral inhibition.

Finally, we wondered if observed differences in SARS-CoV-2 infection course in different hMDMs-HAE co-cultures might be associated with alterations of cytokine and/or chemokine signalling. We analysed the concentration of a wide spectrum of cytokines and chemokines, released into the basolateral medium of the co-cultures using the Luminex, as described in Methods. In this experiment, we consistently detected in all experiments the presence of four chemokines (CXCL1, IL-8, CXCL2, CXCL10/IP-10) and two growth factors (G-CSF and VEGF). The chemokines, CXCL1, CXCL2, IL-8 and CXCL10/IP-10, promote inflammation and antiviral response by the recruitment and activation of neutrophils (60–63), while G-CSF promotes neutrophil expansion and egress from the bone marrow to the bloodstream, resulting in the accumulation of neutrophils at the infection site (64). Moreover, CXCL10/IP-10 chemoattracts CXCR3-positive cells, including macrophages, dendritic cells, NK cells and activated T lymphocytes, toward inflammation sites during infection or neoplastic transformation (63), whereas VEGF both stimulate the migration of macrophages and promote vascular permeability that helps immune cell migration to the infection sites (62). The chemokines seem to play an important role in the immune response of lung tissue, relying on the migration and activation of innate immune cells. They can be constitutively produced by the lung tissue, which is in agreement with the obtained results, as they were detected regardless of the infection status of the HAE. Previously, the CCR2-monocyte axis was suggested to be critical for the virus control and restriction of inflammation within the respiratory tract during SARS-CoV-2 infection; mice lacking CCR2 showed higher viral loads in the lungs, increased lung viral dissemination, and elevated inflammatory cytokine responses (65). Nonetheless, we have detected CCL2 only in one, out of three experiments (**Supplementary Table 1**), thus we cannot conclude about CCR2 role in our model. Other cytokines previously reported to be upregulated during SARS-CoV-2 infection are IFN type I, TNF-α and IL-6 (25, 34, 66, 67). However, none of these cytokines was detected in any of our repetitions except for TNF-α, which was detected in one of the replicates, at low quantity and without statistically significant differences (**Supplementary Table 1**). In agreement with our results, Niles et al. (33) showed a lack of increased expression of TNF-α, IL-6 and type I IFNs in infected MDMs. Additionally, other studies have shown the suppression of the antiviral type I IFNs response in these cells during SARS-CoV-2 infection (26, 32, 68).

Previous reports have shown that HAE secretes a number of pro-inflammatory cytokines and chemokines, such as IL-12p70, IFN-γ, IL-1β, IL-6 and IL-8, in response to SARS-CoV-2 infection (69). In our study, IL-8 was not upregulated during the infection and the levels of other cytokines detected by Djidrovski et al. (69) were below the detection level. Moreover, we did not observe any significant differences between different models of hMDMs-HAE co-cultures. This may suggest that the cytokine/chemokine production is either not responsible for the effect of the macrophages on the course of SARS-CoV-2 infection in the HAE model, or that the cytokine production between HAE donors is so divergent that we could not record a significant difference using the given methodology. What is more, although cytokine production was previously analysed in SARS-CoV-2 infected hMDMs, showing i.e. the pro-inflammatory cytokines TNF-α, IP-10, IFN-γ, IL-6 and IL-8 production in SARS-CoV-2-exposed hMDMs (25, 34, 70), to date, there are no data available on the cytokine production in the hMDMs-lung epithelial cell co-cultures. So far, the relationship between the inflammatory response induced by SARS-CoV-2 replication and immune cells in HAEs was tested only by the addition of PBMC to the basolateral side of the cultures (71) showing that the immune cells strongly affected the inflammatory profile induced by SARS-CoV-2 infection, dampening the production of several immunoregulatory/inflammatory signals (e.g., IL-35, IL-27, and IL-34). In our model, the cytokine repertoire and amount released to the basolateral side of HAE might not be altered also due to the localization of hMDMs at the apical side of HAE, at a far distance from the basolateral medium, in which the concentration of the cytokines was analysed. Thus, we believe that the inhibition of SARS-CoV-2 infection by lung macrophages is mediated by cell-cell contact, not by the cytokines release.

There are several limitations of our study that should be considered. First, the study was performed using *in vitro* differentiated hMDMs, which can have phenotypic variations compared to the ones present *in vivo*. A precise characterisation of AMs is very complex and even though several studies have focused on this (21, 72, 73), there is still not a consented phenotypic characterization of AMs and their different activations states. Although it was shown that AMs can originate from monocytes migrating from the circulation (6, 16, 18), it is important to consider the limitations when using them in experiments. It is also worth mentioning that the co-cultures were performed with HAE and hMDMs from different donors. Even though AMs alone are not able to recognize and reject allogeneic cells without the help of other immune cells, such as activated T cells (74), having co-cultures from autologous cells could better resemble the physiological condition. Another limitation we consider important is the number of samples evaluated for the cytokine analysis. Our cytokine analysis was aiming to measure a broad spectrum of cytokines and chemokines, which limited the number of repetitions we were able to evaluate. Additionally, as we observed, the cytokine production is donor variable and these differences influenced the lack of statistical significance of our results. Evaluation of a selected group of cytokines in a large number of samples can provide more answers regarding the cytokine production during SARS-CoV-2 infection in the airway tissue. The immune response during SARS-CoV-2 is a topic that still has a lot of uncertainties and although several studies have focused on this topic, immune response regulation at the cytokine level both *in vivo* and *in vitro* is still largely unknown.

In conclusion, our results indicate, for the first time, that hMDMs substantially delay SARS-CoV-2 replication and dissemination in lung epithelial cells by close cell-cell contact, in a cytokine-independent manner. We propose that hMDMs viral inhibition is mediated through the phagocytosis of the infected cells, while macrophage infection results in the inhibition of their antiviral activity, led by a yet unknown mechanism.

## Data availability statement

The raw data supporting the conclusions of this article will be made available by the authors, without undue reservation.

## Ethics statement

Ethical review and approval were not required for the study on human participants in accordance with the local legislation and institutional requirements. The patients/participants provided their written informed consent to participate in this study.

## Author contributions

Conceptualization: KP, ML, EB-D, AM. Methodology: EB-D, AG-B, MSu and ZR. Investigation: EB-D, AS, AG-B and MSu. Data curation: EB-D and ML. Data analysis: EB-D, ML, AS and KP. Funding acquisition: KP. Supervision: KP and ML. Writing – original draft: EB-D and ML. Writing – review & editing: KP, ML, MSi and MSa. All authors contributed to the article and approved the submitted version.

## Funding

This work was supported by a subsidy from the Polish Ministry of Science and Higher Education for research on SARS-CoV-2, a grant from the National Science Center (UMO-2017/27/B/NZ6/02488) to KP and by EU-Horizon2020 ITN OrganoVir grant 812673.

## Acknowledgments

We would like to thank Monika Baj-Krzyworzeka (Jagiellonian university Medical College, Krakow, Poland) for macrophage differentiation protocol. The preprint of the manuscript is available here https://doi.org/10.21203/rs.3.rs-1715827/v1.

## Conflict of interest

The authors declare that the research was conducted in the absence of any commercial or financial relationships that could be construed as a potential conflict of interest.

## Publisher’s note

All claims expressed in this article are solely those of the authors and do not necessarily represent those of their affiliated organizations, or those of the publisher, the editors and the reviewers. Any product that may be evaluated in this article, or claim that may be made by its manufacturer, is not guaranteed or endorsed by the publisher.
