# Supplementary material for: The interplay between the airway epithelium and tissue macrophages during the SARS-CoV-2 infection
Source: Front Immunol. 2022 Oct 6;13:991991. doi: 10.3389/fimmu.2022.991991 (PMC9582145; doi:10.3389/fimmu.2022.991991)
Supplement: Supplementary file 1 [file DataSheet_1.pdf]

Supplementary Material

1 Supplementary Figures and Tables

1.1 Supplementary Figures

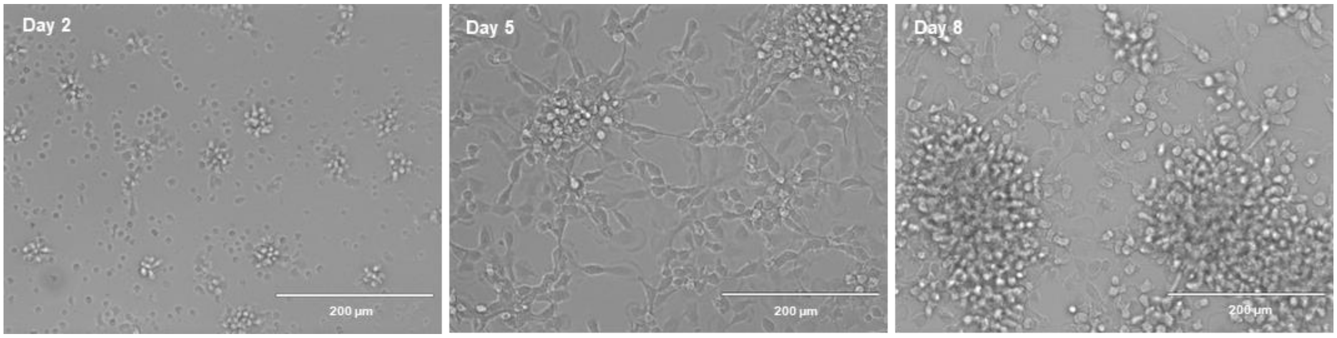

Supplementary Figure 1. Differentiation of hMDMs. Light microscopy images of primary monocytes differentiated to macrophages during 8 days in RPMI medium supplemented with 10% FBS.

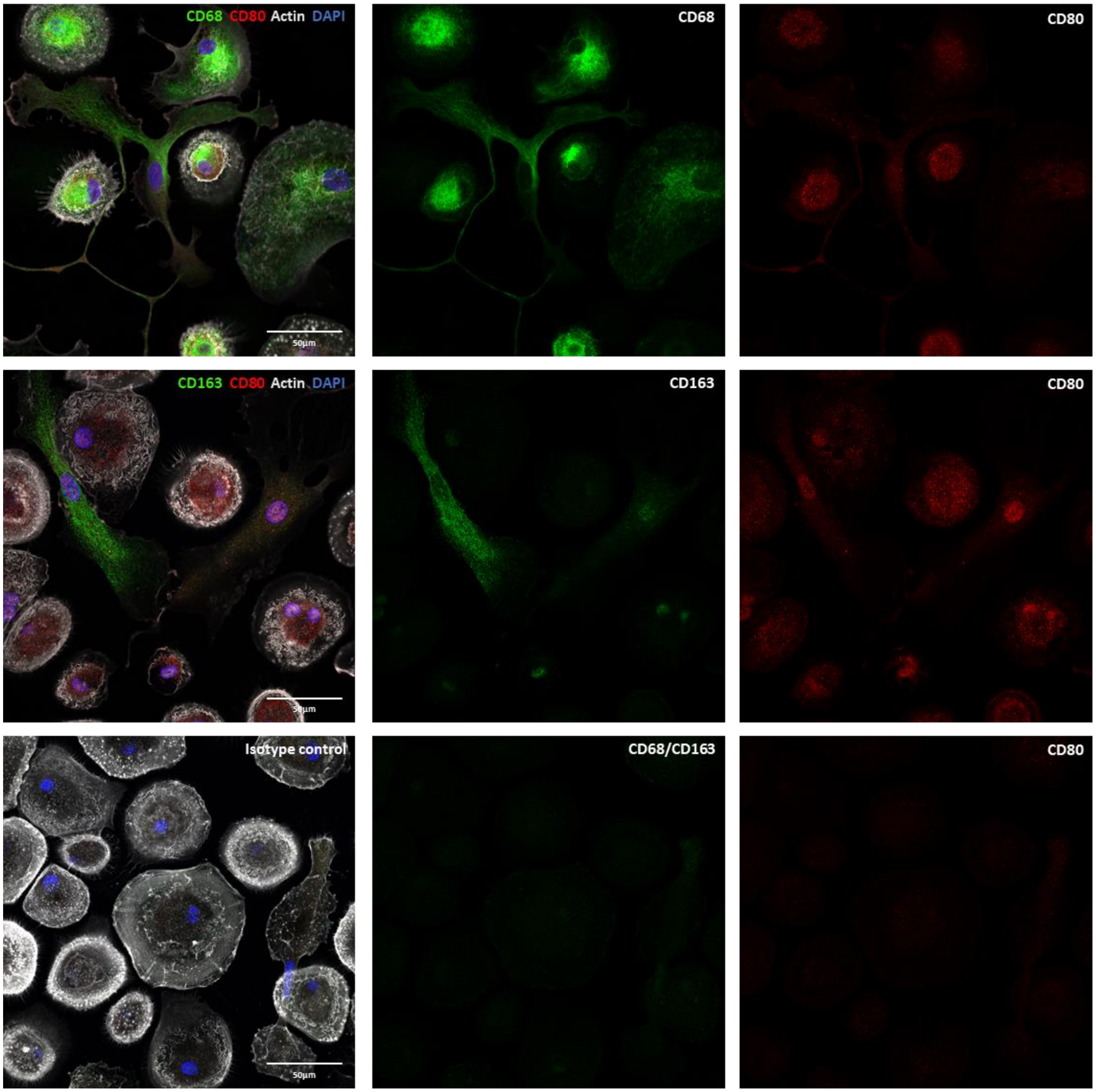

Supplementary Figure 2. hMDMs immunophenotype. Primary human monocytes were differentiated to hMDMs for 10 days and immunostained with differentiation markers CD68, CD163 and CD80. Panels on the left show merged images. CD80 is shown in red, CD68/CD163 is shown in green and actin is shown in white.

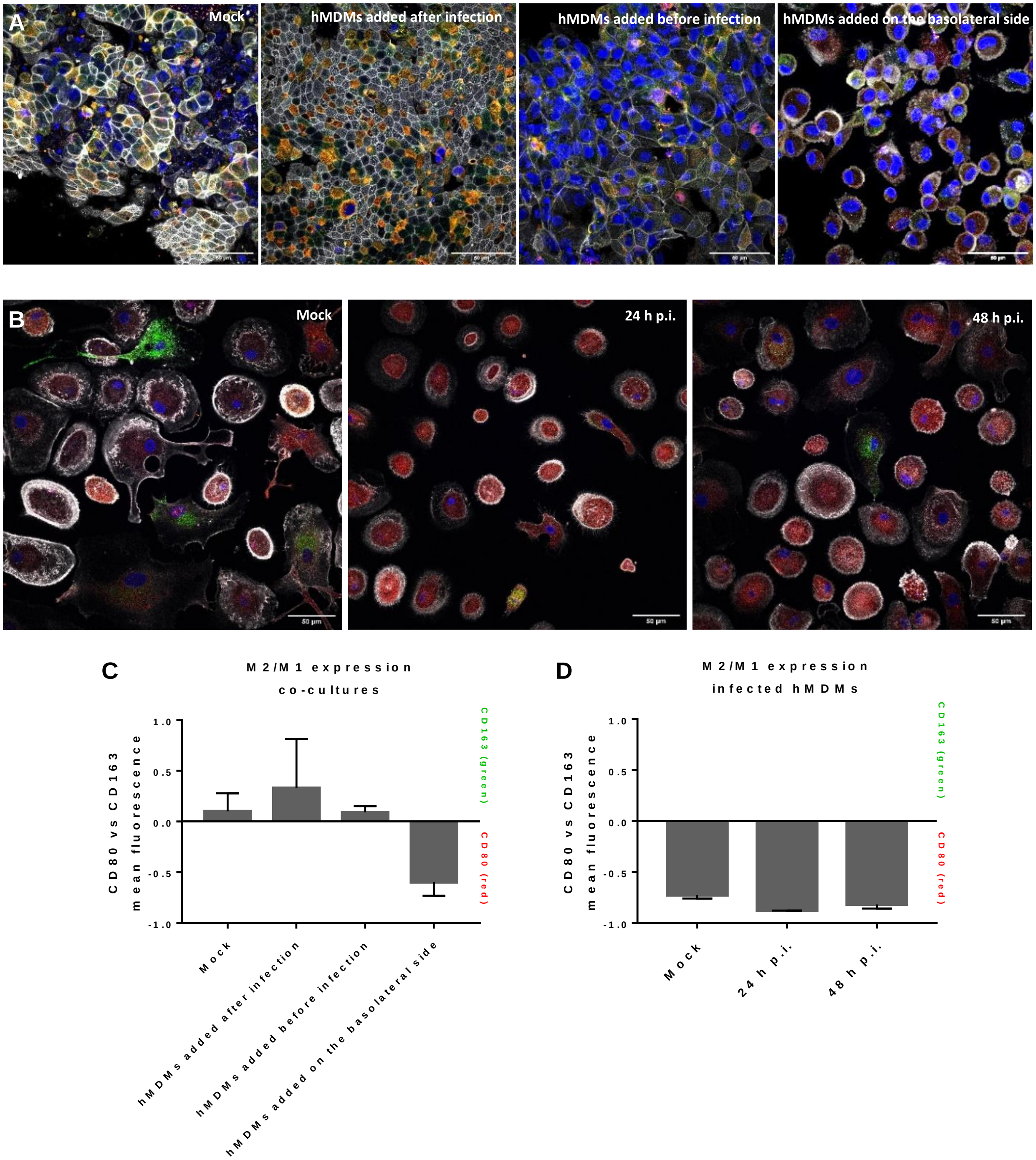

Supplementary Figure 3. Quantification of M2/M1 markers in immunostained images of infected hMDMs and HAE-hMDMs co-cultures. The mean fluorescence of CD163 was calculated in relation to the mean fluorescence of CD80. CD80 is shown in red, CD163 is shown in green, actin is shown in white and nuclei in blue. Positive fluorescence values show a higher expression of CD163 and negative values of CD80. **(A)** Expression of M2/M1 in infected HAE-hMDMs co-cultures 48 h p.i.. **(B)** expression of M2/M1 in infected hMDMs. **(C)** Mean fluorescence of CD163/CD80 for co-cultures (48 h. p.i.). **(D)** Mean fluorescence of CD163/CD80 for infected hMDMs. Means ± SEM are shown. Groups were compared by the Kruskal-Wallis test and Dunn's multiple comparisons test. ** $p<0.01$, ***$p<0.001$.

1.2 Supplementary Tables

Supplementary Table 1. Cytokine analysis results.

				MCP-1	MIP-3a	MIP-1b	GROa (CXCL1)	IP-10	GROb (CXCL2)	IL-8	EGF	FLT-3 Ligand	G-CSF	GM-CSF	IL-17a	IL-1ra	IL-6	IL-7	PDGF-AA	TNF-a	VEGF	IL-1a
1	Exp 1	A. HAE infected first then MDM added	48h	NN	NN	NN	107.47	NN	NN	89.55	NN	NN	5.28	NN	0.51	NN	NN	NN	NN	NN	NN	NN
2	Exp 1	A. HAE infected first then MDM added	48h	NN	NN	NN	116.95	2.73	NN	101.23	NN	3.22	4.12	NN	0.51	NN	NN	NN	NN	NN	NN	NN
3	Exp 1	A. HAE infected first then MDM added	48h	NN	NN	NN	106.05	NN	NN	72.96	NN	NN	NN	NN	NN	NN	NN	NN	NN	NN	NN	NN
4	Exp 1	B. Co-culture infected	48h	NN	NN	NN	127.05	3.53	NN	89.55	NN	3.22	20.34	NN	0.51	NN	NN	NN	NN	NN	NN	NN
5	Exp 1	B. Co-culture infected	48h	NN	NN	NN	145.43	NN	NN	107.04	NN	3.22	17.53	NN	0.51	NN	NN	NN	NN	NN	NN	NN
6	Exp 1	B. Co-culture infected	48h	NN	NN	NN	75.08	0.32	NN	56.70	NN	NN	NN	NN	NN	NN	NN	NN	NN	NN	NN	NN
7	Exp 1	D. Just HAE infected	48h	NN	NN	NN	76.98	NN	NN	46.49	NN	NN	NN	NN	0.51	NN	NN	NN	NN	NN	NN	NN
8	Exp 1	D. Just HAE infected	48h	NN	NN	NN	47.63	NN	NN	13.87	NN	NN	NN	NN	NN	NN	NN	NN	NN	NN	NN	NN
9	Exp 1	D. Just HAE infected	48h	NN	NN	NN	NN	NN	NN	12.42	NN	NN	NN	NN	NN	NN	NN	NN	NN	NN	NN	NN
10	Exp 1	H. Co-culture Mock	48h	NN	NN	NN	111.62	0.90	NN	93.06	NN	1.38	7.50	NN	0.51	NN	NN	NN	NN	NN	NN	NN
11	Exp 1	I. HAE Mock	48h	NN	NN	NN	75.08	2.73	NN	51.39	NN	NN	0.03	NN	0.51	NN	NN	NN	NN	NN	NN	NN
12	Exp 1	A. HAE infected first then MDM added	72h	NN	NN	NN	120.82	1.16	NN	99.31	NN	3.22	20.34	NN	0.51	NN	NN	NN	NN	NN	NN	NN
13	Exp 1	A. HAE infected first then MDM added	72h	NN	NN	NN	149.72	7.42	NN	114.99	NN	6.79	22.18	NN	0.51	NN	NN	NN	NN	NN	NN	NN
14	Exp 1	A. HAE infected first then MDM added	72h	NN	NN	NN	125.82	2.52	NN	79.40	NN	NN	NN	NN	0.51	NN	NN	NN	NN	NN	NN	NN
15	Exp 1	B. Co-culture infected	72h	NN	NN	NN	122.09	9.50	NN	94.18	NN	3.22	10.14	NN	0.51	NN	NN	NN	NN	NN	NN	NN
16	Exp 1	B. Co-culture infected	72h	NN	NN	NN	78.83	0.90	NN	72.33	NN	1.38	25.80	NN	NN	NN	NN	NN	NN	NN	NN	NN
17	Exp 1	B. Co-culture infected	72h	NN	NN	NN	111.62	2.52	NN	100.43	NN	0.42	21.26	NN	0.51	NN	NN	NN	NN	NN	NN	NN
18	Exp 1	D. Just HAE infected	72h	NN	NN	NN	58.05	2.52	NN	47.41	NN	NN	1.60	NN	NN	NN	NN	NN	NN	NN	NN	NN
19	Exp 1	D. Just HAE infected	72h	NN	NN	NN	82.43	4.29	NN	61.03	NN	NN	NN	NN	NN	NN	NN	NN	NN	NN	NN	NN
20	Exp 1	D. Just HAE infected	72h	NN	NN	NN	120.18	5.20	NN	78.62	NN	3.22	20.34	NN	NN	NN	NN	NN	NN	NN	NN	NN
21	Exp 1	H. Co-culture Mock	72h	1.58	NN	NN	128.86	0.63	NN	114.02	NN	3.22	37.07	NN	0.51	NN	NN	NN	NN	NN	NN	NN
22	Exp 1	I. HAE Mock	72h	NN	NN	NN	142.13	7.59	NN	92.74	NN	1.38	NN	NN	0.51	NN	NN	NN	NN	NN	NN	NN
23	Exp 2	A. HAE infected first then MDM added	24h	NN	53.18	NN	2608.00	NN	401.08	NN	413.14	NN	445.50	NN	NN	286.10	NN	NN	NN	NN	162.14	NN
24	Exp 2	A. HAE infected first then MDM added	24h	NN	30.24	NN	2202.00	12.72	302.04	NN	473.48	NN	252.06	NN	NN	257.34	NN	NN	NN	NN	156.20	NN
25	Exp 2	A. HAE infected first then MDM added	24h	NN	32.18	NN	2608.00	NN	259.42	NN	419.90	NN	525.30	NN	NN	176.66	NN	NN	NN	NN	203.74	NN
26	Exp 2	B. Co-culture infected	24h	75.52	232.04	269.88	8148.00	6.42	1026.24	NN	511.06	NN	818.70	37.92	NN	715.80	156.72	NN	49.06	NN	157.98	NN
27	Exp 2	B. Co-culture infected	24h	99.22	81.54	NN	4634.00	106.56	578.86	NN	484.68	NN	290.02	36.20	NN	1106.88	153.06	NN	NN	NN	127.68	NN
28	Exp 2	B. Co-culture infected	24h	44.70	59.40	NN	3704.00	13.76	527.54	NN	487.74	NN	366.20	29.54	NN	1129.92	275.80	NN	NN	NN	209.60	NN
29	Exp 2	D. Just HAE infected	24h	NN	67.02	NN	4268.00	NN	457.04	NN	421.98	NN	392.08	NN	NN	44.64	NN	NN	NN	NN	185.12	NN
30	Exp 2	D. Just HAE infected	24h	NN	296.24	325.02	6060.00	NN	1291.18	NN	426.14	NN	695.18	32.84	NN	255.52	NN	NN	49.06	NN	236.88	NN
31	Exp 2	D. Just HAE infected	24h	12.26	152.08	187.40	6688.00	NN	762.86	NN	517.12	NN	799.82	25.56	NN	246.38	NN	NN	83.60	NN	222.14	NN
32	Exp 2	H. Co-culture Mock	24h	86.86	1149.10	725.58	10886.00	72.02	3786.00	NN	560.40	NN	3774.00	188.44	NN	790.34	357.12	NN	66.44	15.00	393.40	NN
33	Exp 2	H. Co-culture Mock	24h	68.80	430.98	284.38	7380.00	NN	1230.14	NN	435.48	NN	760.70	92.40	NN	717.10	299.36	NN	NN	18.50	164.50	NN
34	Exp 2	I. HAE Mock	24h	16.32	93.90	NN	5532.00	NN	600.26	NN	497.90	NN	709.36	36.20	NN	277.20	310.80	NN	NN	NN	264.16	NN
35	Exp 2	A. HAE infected first then MDM added	48h	NN	NN	NN	171.72	NN	6.59	329.23	38.30	NN	82.00	NN	0.51	NN	NN	NN	NN	0.65	NN	8.64
36	Exp 2	A. HAE infected first then MDM added	48h	NN	NN	NN	328.57	1.41	43.33	451.26	55.63	6.79	522.14	NN	0.51	NN	NN	NN	NN	2.75	2.20	NN
37	Exp 2	A. HAE infected first then MDM added	48h	NN	NN	NN	327.16	NN	34.16	407.80	49.60	6.79	883.57	NN	0.51	NN	NN	0.20	NN	4.17	3.86	NN
38	Exp 2	B. Co-culture infected	48h	NN	NN	NN	253.60	NN	20.06	322.94	44.65	NN	363.50	NN	0.51	NN	NN	NN	NN	0.65	NN	NN
39	Exp 2	B. Co-culture infected	48h	NN	NN	NN	111.62	2.42	NN	178.33	10.93	NN	91.63	NN	0.51	NN	NN	NN	NN	NN	NN	NN
40	Exp 2	B. Co-culture infected	48h	NN	NN	NN	139.34	NN	NN	156.91	10.93	NN	130.91	NN	0.51	NN	NN	NN	NN	NN	NN	NN
41	Exp 2	D. Just HAE infected	48h	NN	NN	NN	256.35	NN	20.06	221.01	37.00	NN	128.12	NN	0.51	NN	NN	NN	NN	NN	0.25	NN
42	Exp 2	D. Just HAE infected	48h	NN	NN	NN	281.37	NN	31.32	295.86	31.70	0.42	185.71	NN	1.15	NN	NN	NN	NN	0.65	0.90	NN
43	Exp 2	D. Just HAE infected	48h	NN	NN	NN	194.16	NN	26.79	205.34	41.50	NN	326.19	NN	0.51	NN	NN	NN	NN	0.65	NN	NN
44	Exp 2	H. Co-culture Mock	48h	NN	NN	NN	361.78	NN	44.56	467.63	38.30	8.55	724.92	NN	0.51	NN	NN	NN	NN	3.46	10.91	5.93
45	Exp 2	H. Co-culture Mock	48h	NN	NN	NN	274.96	NN	31.32	382.80	34.37	1.38	232.40	NN	0.51	NN	NN	0.20	NN	1.34	NN	4.44
46	Exp 2	I. HAE Mock	48h	NN	NN	NN	352.22	NN	29.84	332.49	59.18	3.22	191.64	NN	0.51	NN	NN	NN	NN	0.65	4.19	NN
47	Exp 2	A. HAE infected first then MDM added	72h	NN	NN	NN	155.98	NN	NN	302.14	33.04	NN	56.65	NN	0.51	NN	NN	NN	NN	NN	NN	9.90
48	Exp 2	A. HAE infected first then MDM added	72h	NN	NN	NN	462.48	1.87	67.52	477.83	100.06	13.76	951.68	NN	1.15	NN	NN	0.66	NN	5.59	11.59	0.81
49	Exp 2	A. HAE infected first then MDM added	72h	NN	NN	NN	296.01	NN	32.75	274.90	33.71	1.38	600.28	NN	0.51	NN	NN	NN	NN	2.04	NN	NN
50	Exp 2	B. Co-culture infected	72h	NN	NN	NN	212.28	NN	14.17	214.30	24.78	NN	486.50	NN	0.51	NN	NN	NN	NN	1.34	NN	NN
51	Exp 2	B. Co-culture infected	72h	NN	NN	NN	201.74	7.91	14.17	316.48	30.34	NN	421.39	NN	NN	NN	NN	NN	NN	1.34	NN	NN
52	Exp 2	B. Co-culture infected	72h	NN	NN	NN	208.68	4.84	9.45	290.73	37.00	NN	187.03	NN	0.51	NN	NN	NN	NN	NN	NN	NN
53	Exp 2	D. Just HAE infected	72h	NN	NN	NN	328.57	NN	32.75	329.23	43.40	1.38	224.72	NN	0.51	NN	NN	NN	NN	0.65	3.53	NN
54	Exp 2	D. Just HAE infected	72h	NN	NN	NN	355.16	1.41	36.90	375.08	38.30	5.02	444.69	NN	1.15	NN	NN	NN	NN	1.34	4.19	NN
55	Exp 2	D. Just HAE infected	72h	NN	NN	NN	302.97	NN	39.53	309.01	42.14	5.02	1037.68	NN	0.51	NN	NN	NN	NN	4.88	2.20	NN
56	Exp 2	H. Co-culture Mock	72h	NN	NN	NN	163.02	NN	29.84	203.77	7.37	NN	459.17	NN	0.51	NN	NN	0.20	NN	1.34	NN	NN
57	Exp 2	H. Co-culture Mock	72h	NN	NN	NN	192.88	NN	9.45	275.55	26.19	NN	145.06	NN	0.51	NN	NN	NN	NN	NN	NN	0.81
58	Exp 2	I. HAE Mock	72h	NN	NN	NN	98.73	NN	NN	119.72	23.34	NN	51.07	NN	0.51	NN	NN	NN	NN	NN	NN	NN
59	Exp 3	A. HAE infected first then MDM added	24h	1075.66	742.08	187.40	12042.00	62.72	547.52	NN	NN	NN	270.46	132.02	NN	453.14	NN	NN	315.12	NN	469.00	NN
60	Exp 3	A. HAE infected first then MDM added	24h	734.66	722.38	222.74	9120.00	49.94	538.82	NN	NN	NN	285.20	124.06	NN	585.70	NN	NN	262.48	NN	410.52	NN
61	Exp 3	A. HAE infected first then MDM added	24h	1137.12	801.48	168.16	19098.00	43.16	610.82	NN	NN	NN	386.62	321.30	NN	376.64	NN	NN	201.24	NN	460.92	NN
62	Exp 3	B. Co-culture infected	24h	1601.98	419.82	205.54	7954.00	190.00	764.76	NN	NN	NN	504.48	619.52	NN	591.34	NN	NN	100.46	NN	273.12	NN
63	Exp 3	B. Co-culture infected	24h	734.96	547.84	NN	8340.00	38.10	528.30	NN	NN	NN	374.70	108.18	NN	70.92	NN	NN	275.28	NN	294.22	NN
64	Exp 3	B. Co-culture infected	24h	1408.76	478.12	NN	6060.00	70.78	639.80	NN	NN	NN	461.26	242.90	NN	161.14	NN	NN	179.36	NN	293.76	NN
65	Exp 3	D. Just HAE infected	24h	1785.84	611.44	254.82	11174.00	92.82	724.22	NN	NN	NN	465.40	374.52	NN	172.78	NN	NN	242.74	NN	326.28	NN
66	Exp 3	D. Just HAE infected	24h	1736.78	580.38	239.12	11464.00	58.42	690.52	NN	NN	NN	408.94	379.96	NN	147.46	NN	NN	242.74	NN	299.16	NN
67	Exp 3	D. Just HAE infected	24h	484.80	559.26	NN	8868.00	33.90	508.08	NN	NN	NN	403.62	31.18	NN	78.66	NN	NN	318.08	NN	292.86	NN
68	Exp 3	H. Co-culture Mock	24h	708.60	482.84	NN	8068.00	23.08	440.76	NN	NN	NN	187.66	119.10	NN	370.04	NN	NN	222.36	NN	373.54	NN
69	Exp 3	H. Co-culture Mock	24h	461.78	426.78	NN	6802.00	19.22	386.50	NN	NN	NN	187.66	59.62	NN	178.58	NN	NN	179.36	NN	315.92	NN
70	Exp 3	I. HAE Mock	24h	859.86	477.14	187.40	8042.00	62.26	556.86	NN	NN	NN	378.30	102.24	NN	113.98	NN	NN	268.90	NN	255.06	NN
71	Exp 3	I. HAE Mock	24h	1370.40	470.24	254.82	11742.00	80.64	692.38	NN	NN	NN	644.40	237.12	NN	235.32	72.26	NN	222.36	NN	294.22	NN
72	Exp 3	A. HAE infected first then MDM added	48h	273.24	298.74	168.16	5126.00	23.90	302.96	NN	18.54	NN	137.94	90.42	NN	534.14	NN	NN	141.08	NN	249.72	NN
73	Exp 3	A. HAE infected first then MDM added	48h	79.10	110.66	NN	2172.00	8.40	148.92	NN	NN	NN	137.94	29.54	NN	248.22	NN	NN	NN	NN	125.12	NN
74	Exp 3	A. HAE infected first then MDM added	48h	360.84	394.72	NN	5728.00	22.06	427.14	NN	NN	NN	231.72	195.32	NN	312.44	NN	NN	92.06	NN	269.84	NN
75	Exp 3	B. Co-culture infected	48h	435.60	273.72	NN	5848.00	55.46	407.50	NN	NN	NN	NN	37.92	NN	NN	NN	NN	49.06	NN	191.78	NN
76	Exp 3	B. Co-culture infected	48h	726.74	496.38	168.16	9946.00	69.40	665.80	NN	NN	NN	64.82	137.98	NN	41.04	NN	NN	92.06	NN	192.88	NN
77	Exp 3	B. Co-culture infected	48h	554.66	465.60	168.16	10770.00	53.96	619.92	NN	NN	NN	297.60	126.06	NN	110.04	NN	NN	186.74	NN	199.42	NN
78	Exp 3	D. Just HAE infected	48h	982.90	451.34	205.54	8490.00	68.24	724.22	NN	NN	NN	402.10	263.00	NN	90.36	NN	NN	104.62	NN	212.24	NN
79	Exp 3	D. Just HAE infected	48h	953.82	383.96	NN	7896.00	49.72	656.84	NN	NN	NN	350.88	251.54	NN	55.70	NN	NN	104.62	NN	189.56	NN
80	Exp 3	D. Just HAE infected	48h	267.82	510.94	NN	7380.00	42.84	535.46	NN	NN	NN	NN	24.78	NN	NN	NN	NN	179.36	NN	202.66	NN
81	Exp 3	H. Co-culture Mock	48h	215.82	300.00	NN	5086.00	11.36	330.84	NN	NN	NN	116.40	76.78	NN	199.66	NN	NN	108.76	NN	202.66	NN
82	Exp 3	H. Co-culture Mock	48h	254.86	346.52	NN	6348.00	11.92	394.08	NN	NN	NN	202.70	50.36	NN	277.20	NN	NN	152.80	NN	233.86	NN
83	Exp 3	I. HAE Mock	48h	388.58	360.18	NN	5976.00	29.34	478.22	NN	NN	NN	351.72	64.32	NN	NN	NN	NN	133.14	NN	163.32	NN
84	Exp 3	I. HAE Mock	48h	741.68	359.08	214.24	9050.00	42.20	606.06	NN	NN	NN	562.90	165.76	NN	51.98	NN	NN	116.98	NN	201.58	NN
85	Exp 3	A. HAE infected first then MDM added	72h	305.76	392.24	NN	5320.00	26.76	366.78	NN	NN	NN	221.72	36.20	NN	273.60	NN	NN	116.98	NN	215.38	NN
86	Exp 3	A. HAE infected first then MDM added	72h	251.48	284.86	NN	4736.00	20.10	328.58	NN	NN	NN	298.06	34.52	NN	268.22	NN	NN	49.06	NN	169.16	NN
87	Exp 3	A. HAE infected first then MDM added	72h	369.14	447.76	NN	6734.00	31.44	491.24	NN	NN	NN	290.48	120.10	NN	211.02	NN	NN	92.06	NN	209.60	NN
88	Exp 3	B. Co-culture infected	72h	704.84	337.74	168.16	6122.00	107.32	646.66	NN	NN	NN	506.74	473.34	NN	298.46	NN	NN	NN	NN	162.14	NN
89	Exp 3	B. Co-culture infected	72h	330.00	463.16	NN	7336.00	44.62	517.40	NN	NN	NN	495.66	83.58	NN	30.56	NN	NN	116.98	NN	190.68	NN
90	Exp 3	B. Co-culture infected	72h	295.00	356.48	NN	5136.00	13.48	364.70	NN	NN	NN	212.04	76.78	NN	NN	NN	NN	53.42	NN	92.92	NN
91	Exp 3	D. Just HAE infected	72h	826.40	423.06	187.40	6240.00	39.92	627.60	NN	NN	NN	479.68	197.28	NN	17.66	NN	NN	31.60	NN	176.06	NN
92	Exp 3	D. Just HAE infected	72h	207.76	485.58	NN	6358.00	517.66	440.32	NN	NN	NN	431.14	46.74	NN	NN	NN	NN	156.66	NN	196.16	NN
93	Exp 3	D. Just HAE infected	72h	881.50	475.66	NN	7716.00	158.14	648.76	NN	NN	NN	471.90	249.62	NN	28.88	NN	NN	108.76	NN	176.06	NN
94	Exp 3	H. Co-culture Mock	72h	290.00	417.50	NN	5994.00	17.96	407.02	NN	NN	NN	230.62	59.62	NN	141.58	NN	NN	121.06	NN	192.88	NN
95	Exp 3	H. Co-culture Mock	72h	221.16	409.00	NN	6058.00	16.48	394.34	NN	NN	NN	232.26	32.84	NN	129.76	NN	NN	108.76	NN	174.92	NN
96	Exp 3	I. HAE Mock	72h	337.78	349.34	NN	4796.00	36.56	413.36	NN	NN	NN	361.70	57.74	NN	NN	NN	NN	57.76	NN	140.32	NN
97	Exp 3	I. HAE Mock	72h	669.02	351.70	NN	5532.00	31.34	497.64	NN	NN	NN	581.08	155.86	NN	41.04	NN	NN	49.06	NN	186.22	NN

*** Cytokine levels were measured by Luminex analysis in pg/ml. NN are samples below the detection level. Each experiment represents repetitions done with different cell donors.**

*** Measured cytokines that were not detected in any experiment: PD-L1, Eotaxin, MIP-3b, MIP-1a, CD40 Ligand, Granzyme B, IFN-a, IFN-b, IFN-g, IL-10, IL-12p70, IL-13, IL-15, IL-17e, IL-1b, IL-2, IL-3, IL-33, IL-4, IL-5, PDGF-AB/BB, RANTES, TGF-a, TRAIL, FGF basic, Fractalkine.**
